# Acquired Brown Syndrome in Head Trauma: Does Fixation of Associated Nasal and Frontal Bone Fractures Provide a Cure?

**DOI:** 10.22599/bioj.144

**Published:** 2020-01-30

**Authors:** Mark Watts, Joe McQuillan, Simon Holmes

**Affiliations:** 1 Royal Devon and Exeter NHS Foundation Trust, GB; 2 Barts Health NHS Trust, GB

**Keywords:** Brown syndrome, trauma, maxillofacial, surgery, resolution

## Abstract

A 43-year-old gentleman presented with vertical double vision following nasal and frontal bone fractures resulting from blunt trauma to the glabella. Orthoptic assessment revealed a diagnosis of traumatic Brown syndrome affecting the right eye. The fractures were fixed with open reduction internal fixation via a coronal flap nine days after the injury was sustained. Evidence of resolution of the syndrome became apparent clinically within 15 days following surgery, which was confirmed with a later orthoptic evaluation. This case demonstrates that prompt surgical intervention of fractures associated with traumatic Brown syndrome may lead to resolution without the need to resort to extraocular muscle surgery.

## Introduction

Brown syndrome is a recognized but rare disorder of ocular motility. Most notably, it presents as a restriction of active and passive elevation of the eye in adduction.

Brown syndrome may be congenital or acquired and is due to a restriction of free movement of the superior oblique tendon (SOT) through the trochlea (a cartilaginous structure attached to the superior nasal aspect of the frontal bone) (Wilson, Eustace & Parks 1989).

Brown syndrome may be acquired from trauma, surgery or inflammation (NORD 2003; Ansons & Davis 2001). Acquired Brown syndrome may be treated conservatively with good clinical outcomes. Depending on the aetiology, treatment options include steroids, non-steroidal anti-inflammatory medication or surgery to the appropriate extra-ocular muscles, or the SOT (AAPOS 2017; Paediatric Ophthalmic Consultants 2014).

## Case report

A 43-year-old male waiter was hit in the glabella region with a bottle by a customer.

**Figure 1:**
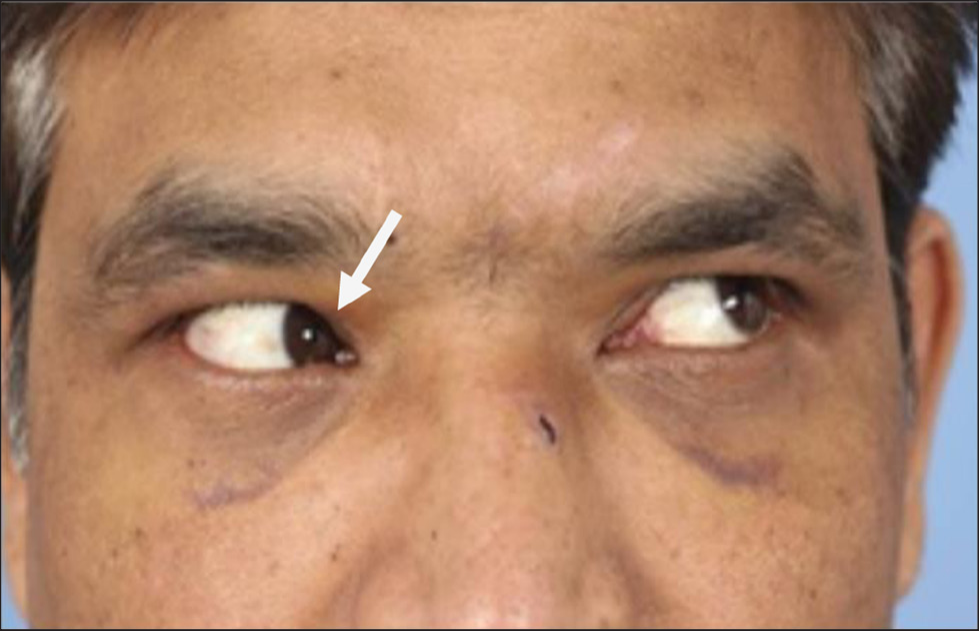
Pre-operative photo of upper left gaze on the day of surgery – The elevation of the right eye is restricted in adduction (see arrow). *Published with the patient’s consent.*

He sustained a depressed skull fracture of the frontal and nasal bones, classified as a ‘bi-lateral Markowitz type 1’ nasoorbitoethmoidal fracture (Markowitz et al. 1991). The fracture extended through to the anterior cranial fossa and cribiform plate. He presented with double vision on upward gaze and on gaze up and left. On examination there was restriction of elevation of the right eye in adduction (Figure 1).

A pre-operative orthoptic assessment (Hess chart [Appendix B.1]), field of binocular single vision (BSV) (Appendix A.1) and motility testing in the nine cardinal views of gaze (Appendix C), confirmed a right traumatic Brown syndrome.

A neuro-ophthalmology examination was otherwise unremarkable, with normal optic nerve function (normal visual acuity, pupillary reactions, fundus examination, colour vision and visual fields).

Surgical access was obtained via a coronal flap, retracted down to the supra-orbital margins.

A step in the frontal bone superior to the nasofrontal suture was identified, reduced and fixed with a 1.3 mm ‘Y’ plate and 6 mm screws. The nasal bone fractures were exposed para-nasally through intra-oral incisions and fixed with two titanium plates with 4 mm screws. The nose was then stabilised with a splint and intra-nasal packs. There was no intra-orbital intervention.

Fifteen days post-operatively, the patient was symptomatically better with a reduction in the severity of diplopia and restriction of elevation of the right eye in adduction.

**Figure 2:**
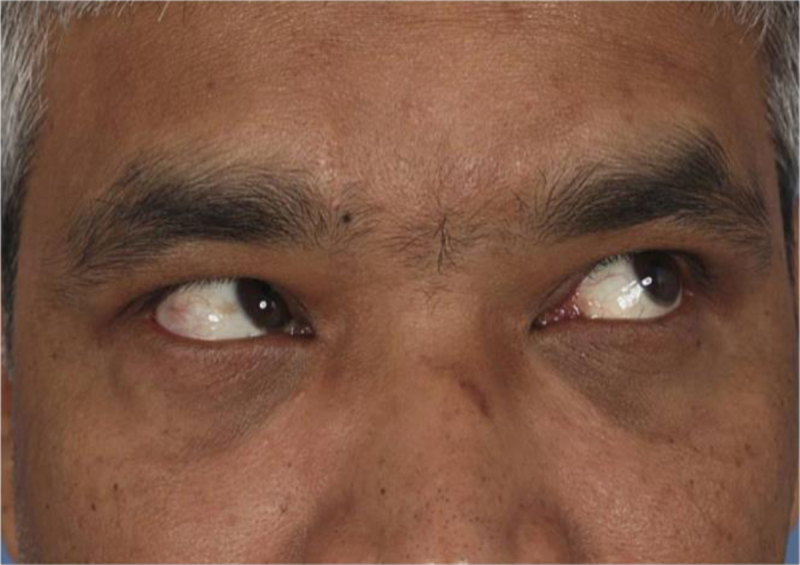
Post-operative photo of ‘upper left gaze’ **–** There is reduced restriction of elevation in adduction of the right eye. *Published with the patient’s consent.*

Orthoptic assessment carried out seven weeks post-operatively found that the extra-ocular motor function had returned to normal, as seen on the post-operative Hess chart (Appendix B.2) and post-operative clinical photographs (Figure 2). Additionally, the area of double vision had resolved (Appendix A.2).

## Discussion

A literature search (Online: PubMed, MEDLINE & Google Scholar) found no reports of attempted correction of traumatic fracture-induced Brown syndrome from open reduction, internal fixation of frontal and nasal bone fractures. Brown syndrome as a result of facial trauma is rare, with no reports of how such cases were managed.

A study of 85 patients with Brown syndrome (38 congenital onsets and 47 acquired) reported spontaneous resolution in 5 of 85 patients (6%). All five were non-traumatic acquired Brown syndrome cases and recovery occurred over a 6- to 48-month period (Wright 1999). The above study supports the fact that repositioning fractured frontal and nasal bone segments to their correct anatomical position, stabilised using titanium plates, in our case of traumatic Brown syndrome, may be responsible for its resolution.

It is possible that the fractures may have restricted the motility of the superior oblique tendon through the trochlea, causing the Brown syndrome pattern of limitation of elevation in adduction and that surgical reduction of the fractures effected a cure. Though it is possible that the Brown syndrome may have resolved spontaneously, it is equally possible that failure to promptly intervene may have heralded a permanent limitation of elevation in adduction as scarring set in. In addition, there are no case reports of spontaneous resolution of traumatic Brown syndrome associated with frontal and nasal bone fractures.

## Conclusion

To our knowledge, this is the first case report of traumatic Brown syndrome where prompt clinical assessment and surgical management of fractures of the frontal and nasal bones was associated with resolution of the syndrome, without the need to resort to extraocular muscle surgery.

## Additional Files

The additional files for this article can be found as follows:

10.22599/bioj.144.s1 Appendix A.1. Pre-operative – Field of Binocular Single Vision.

10.22599/bioj.144.s2 Appendix A.2. Post-operative – Field of Binocular Single Vision.

10.22599/bioj.144.s3 Appendix B.1. Pre-operative HESS chart.

10.22599/bioj.144.s4 Appendix B.2. Post-operative HESS chart.

10.22599/bioj.144.s5 Appendix C. Pre-operative photographs of the patient in the nine cardinal positions of gaze – taken on the day of surgery.

## References

[1] American Association for Paediatric Ophthalmology and Strabismus (AAPOS). 2017 Brown Syndrome. Available at https://www.aapos.org/terms/conditions/29 [Last accessed: 06/08/2017].

[2] Ansons, A and Davis, H. 2001 Diagnosis and Management of Ocular Motility Disorders, 3^rd^ edn, 431 Oxford: Blackwell Science Ltd DOI: 10.1002/9780470698839

[3] Markowitz, BL, Manson, PN, Sargent, L, et al. 1991 Management of the medial canthal tendon in nasoethmoid orbital fractures: the importance of the central fragment in classification and treatment. Plast Reconstr Surg, 87(5): 843–53. DOI: 10.1097/00006534-199105000-00005 2017492

[4] National Organisation for Rare Disorders (NORD). 2003 Brown Syndrome. Available at https://rarediseases.org/rare-diseases/brown-syndrome/ [Last accessed: 06/08/2017].

[5] Paediatric Ophthalmic Consultants. 2014 Complex Strabismus & Syndromes New York Available at http://www.pedseye.com/strabismus_syndromes.htm [Last accessed: 06/08/2017].

[6] Wilson, M, Eustis, H, Jr and Parks, M. 1989 Brown’s syndrome (Major Review). Survey of Ophthalmology, 34: 153–172. DOI: 10.1016/0039-6257(89)90100-8 2694414

[7] Wright, KW. 1999 Brown’s syndrome: diagnosis and management. Trans Am Ophthalmol Soc, 97: 1023–1109. 10703149 PMC1298285

