# Supplementary material for: Acquired Brown Syndrome in Head Trauma: Does Fixation of Associated Nasal and Frontal Bone Fractures Provide a Cure?
Source: Br Ir Orthopt J. 2020 Jan 30;16(1):1–3. doi: 10.22599/bioj.144 (PMC7510388; doi:10.22599/bioj.144)

A.1 **Pre-operative – Field of Binocular Single Vision**

The ‘hatched’ area in black ink, demonstrates the area of diplopia (double vision)

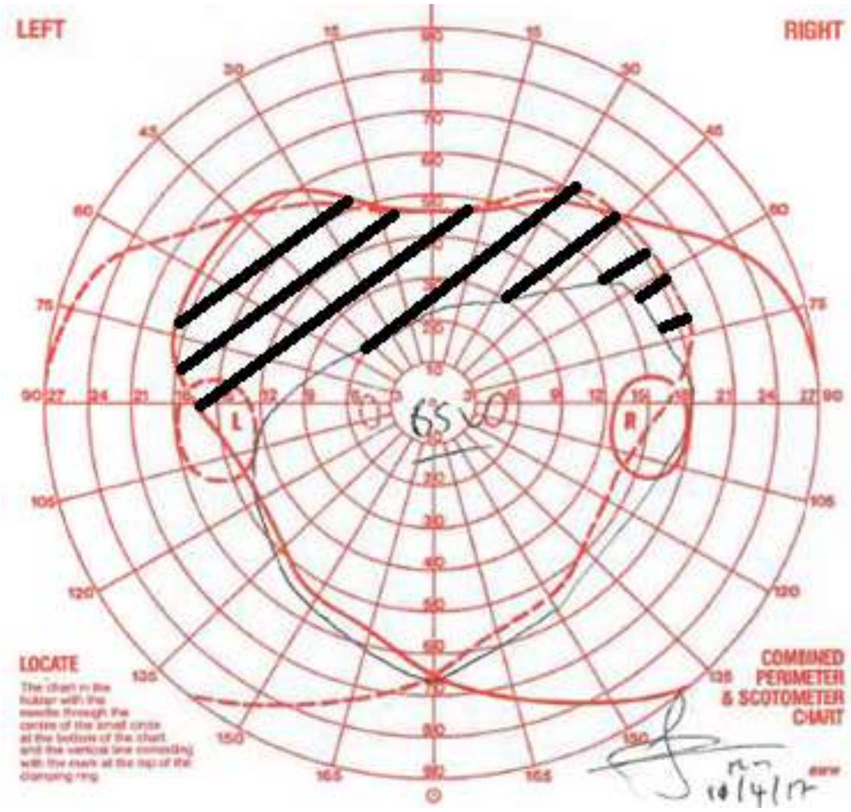

Supplement: Appendix A.1. — Pre-operative – Field of Binocular Single Vision. [file bioj-16-1-144-s1.pdf]
