# Supplementary material for: Acquired Brown Syndrome in Head Trauma: Does Fixation of Associated Nasal and Frontal Bone Fractures Provide a Cure?
Source: Br Ir Orthopt J. 2020 Jan 30;16(1):1–3. doi: 10.22599/bioj.144 (PMC7510388; doi:10.22599/bioj.144)

B.1 **Pre-operative HESS chart.** The restriction of elevation of the right eye in **ad**duction (red arrow) and the left eye overcompensating with excessive elevation in **ab**duction (green arrow).

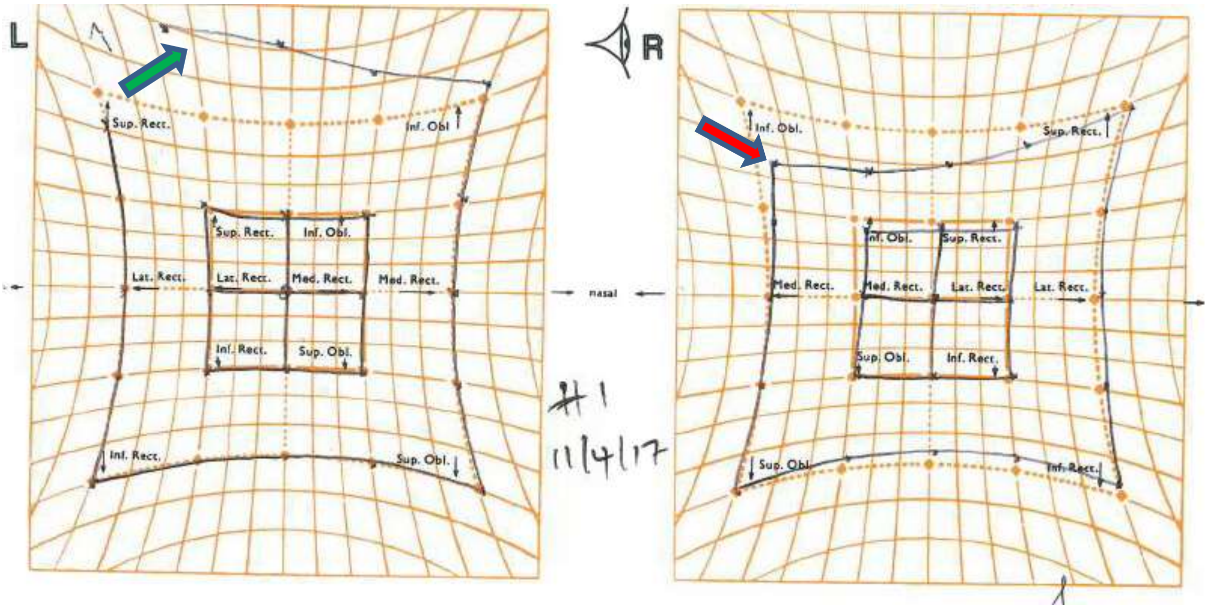

Supplement: Appendix B.1. — Pre-operative HESS chart. [file bioj-16-1-144-s3.pdf]
