# Supplementary figures and images for: Acquired Brown Syndrome in Head Trauma: Does Fixation of Associated Nasal and Frontal Bone Fractures Provide a Cure?
Source: Br Ir Orthopt J. 2020 Jan 30;16(1):1–3. doi: 10.22599/bioj.144 (PMC7510388; doi:10.22599/bioj.144)

## B.2 Post-operative HESS chart. Restoration of normal extraocular muscle motility.

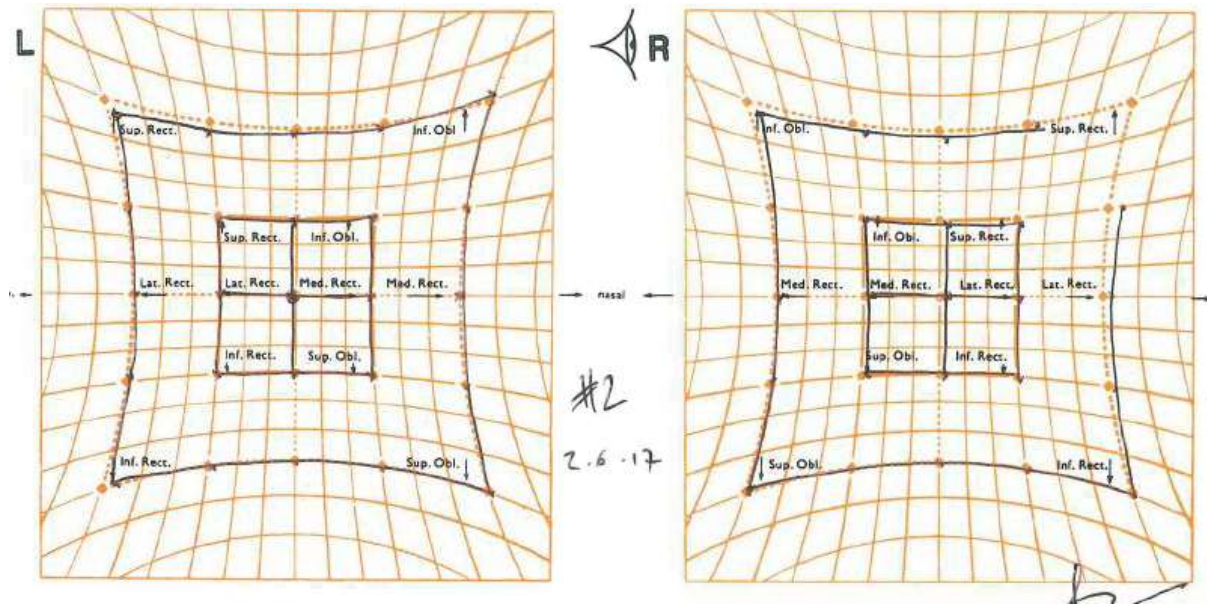

Supplement: Appendix B.2. — Post-operative HESS chart. [file bioj-16-1-144-s4.pdf]
