# Supplementary material for: Acquired Brown Syndrome in Head Trauma: Does Fixation of Associated Nasal and Frontal Bone Fractures Provide a Cure?
Source: Br Ir Orthopt J. 2020 Jan 30;16(1):1–3. doi: 10.22599/bioj.144 (PMC7510388; doi:10.22599/bioj.144)

C Pre-operative photographs of the patient in the nine cardinal positions of gaze – taken on the day of surgery. On gazing to the upper left, the right eye's elevation is restricted in adduction (see arrow). This is a diagnostic signs of Brown's syndrome.
Published with the patient's consent.

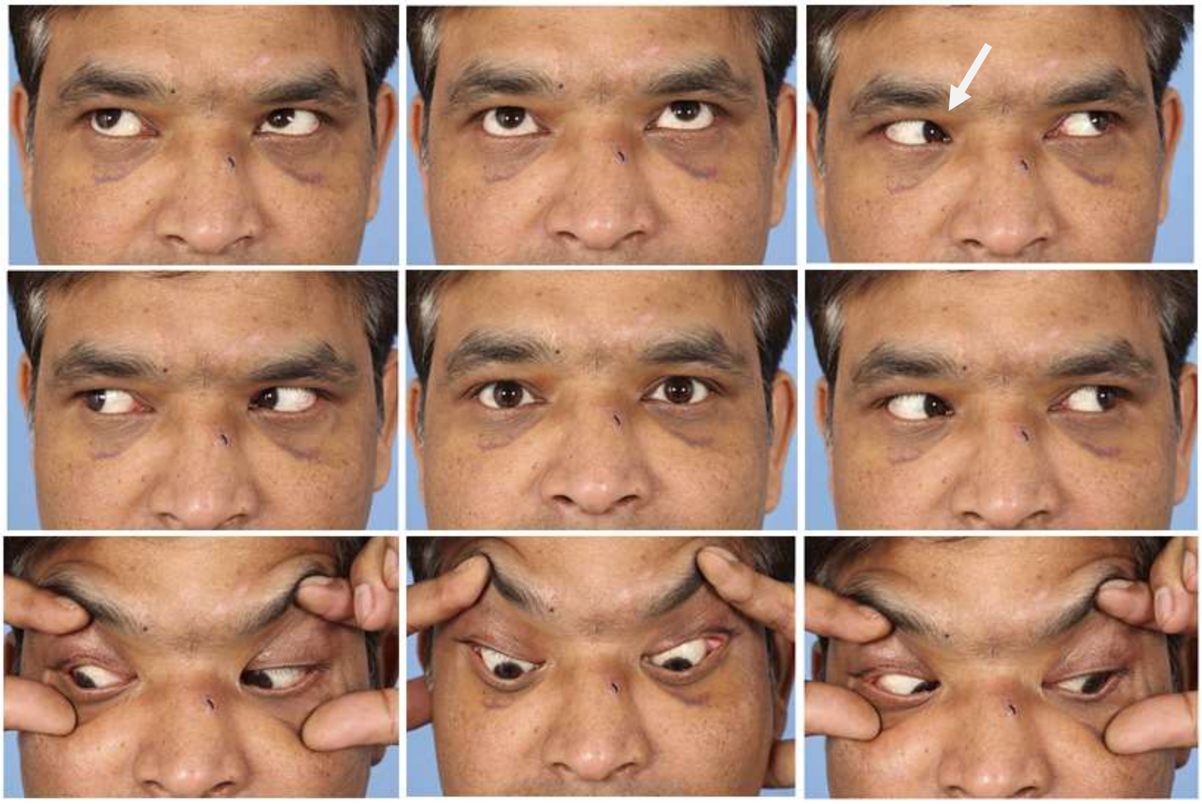

Supplement: Appendix C. — Pre-operative photographs of the patient in the nine cardinal positions of gaze – taken on the day of surgery. [file bioj-16-1-144-s5.pdf]
